# Too Sensitive to Fail: The Impact of Sentiment Connectedness on Stock Price Crash Risk

**DOI:** 10.3390/e27040345

**Published:** 2025-03-27

**Authors:** Jie Cao, Guoqing He, Yaping Jiao

**Affiliations:** 1 School of Economics and Management, Changsha University of Science and Technology, Changsha 410076, China; 2 School of Mathematics and Statistics, Changsha University of Science and Technology, Changsha 410076, China; guoqinghe824@163.com (G.H.); jiaoyp_1116@163.com (Y.J.)

**Keywords:** investor sentiment spillover, sentiment connectedness, stock price crash risk

## Abstract

Using a sample of S&P 500 stocks, this paper examines the investor sentiment spillover network between firms and assesses how the sentiment connectedness in the network impacts stock price crash risk. We demonstrate that firms with higher sentiment connectedness are more likely to crash as they spread more irrational sentiment signals and are more sensitive to investor behaviors. Notably, we find that the effect of investor sentiment on crash risk mainly stems from sentiment connectedness among firms rather than firms’ individual sentiment, especially when market sentiment is surging or declining. These findings remain robust after controlling for other determinants of crash risk, including stock price synchronicity, accounting conservatism, and internal corporate governance strength. Our results underscore the importance of sentiment connectedness among firms and provide valuable insights for risk management among investors and regulatory authorities involved in monitoring risk.

## 1. Introduction

Investor sentiment is widely recognized as a critical factor behind asset price fluctuations [1,2,3,4]. Recent financial uncertainties and economic vulnerabilities have led to increasing sensitivity and widespread investor sentiment and panic dissemination, resulting in severe economic consequences [5,6]. For example, the failure of Silicon Valley Bank on March 10, 2023, has raised significant concerns about broader banking stability. Consequently, investors initiated a sell-off of related stocks, causing sharp declines in over 20 banking equities by March 13, 2023. The collapse of Silicon Valley Bank and subsequent market concerns have continued to draw attention from investors and regulators regarding the transmission of investor sentiment. Another example demonstrating the interconnected nature of investor sentiment is the panic selling induced by recent geopolitical conflicts [7,8,9]. These cases vividly illustrate that financial markets are susceptible to sentiment and its connectedness. Collective panic among investors, driven by market information, herd behavior, and sector-specific emotional transmission, causes instability in financial markets [10,11]. Therefore, studying the impact of investor sentiment connectedness on stock prices is crucial.

This paper considers the connectedness of investor sentiment between firms and evaluates the impact of sentiment connectedness on firm crash risk. Baker and Wurgler (2007) [6] have confirmed that stock price crash risk may be driven by investor sentiment, which is often regarded as factors beyond the company fundamentals (e.g., liquidity [12], book-to-market ratio [13]). West (1988) [14] and Shiller (1990) [15] argue that the irrational emotions of investors, such as “enthusiasm” or “panic”, have the potential to trigger fluctuations and bubbles in asset prices. In light of this, some studies have shown the relationship between investor sentiment and crash risk, such as [16,17,18,19]. While these viewpoints can confirm the relationship between investor sentiment and stock price collapses, they often overlook the connectedness of investor sentiments, which can sometimes hinder the accurate identification of the impact of investor sentiment. The connectedness phenomenon indicates that sentiment shock can spread from one firm to another through financial linkages [20]. Indeed, investor sentiment has been shown to be sensitive to external influences and exhibit spillover across firms in financial markets [6,20,21]. This means that the sentiment of one firm is not solely influenced by its own sentiment, but it can also be significantly impacted by the sentiments of other firms.

This paper uses the nonlinear Granger causality method [22] to study the sentiment spillover network among S&P 500 stocks. The sentiment spillover network differs from previous financial linkages based on business connections [23,24,25] or price co-movements [22,26,27]. First, the firm sentiment connectedness is driven by the exogenous investor sentiment factor, often regarded as irrational and weakly related to company fundamentals [6]. Second, the sentiment connectedness of a firm cannot be entirely attributed to its financial characteristics. For instance, in the constructed sentiment spillover network, *Amazon* is linked to many other firms, whereas *JP Morgan Chase* and *Goldman Sachs* have much less sentiment connectedness despite being ranked as global systemically important financial institutions. Taken together, our findings suggest that the investor sentiment of a firm can receive from or spread to other firms, even without directly affecting ownership or asset flows.

We measure the sentiment connectedness of a firm based on different network centrality measurements. According to El-Khatib et al. (2015) [24], the connectedness of an individual in a network assumes nodes (1) are linked to many other firms; (2) are close to other individuals; (3) are located on the path connecting any other pairs of individuals; and (4) are linked to other highly linked individuals. Motivated by this, many studies have shown the structure and impact of financial networks using degree centrality [28,29,30], closeness centrality [24,31], betweenness centrality [32], eigenvector centrality [33,34], and so on. These indexes measure the centrality or extent of a firm’s connectedness with others and the possible information and resource exchange [35]. However, these centralities only consider one kind of network linkage and influence, causing bias and violating measures of network relationships [36]; sometimes, these centralities are intrinsically relevant [37,38]. To eliminate such biases and measure different influences of a node, we aggregate different network centrality indexes based on the entropy weight method [39] and propose a novel firm sentiment connectedness index, which is referred to as FirmSentix, to measure the connection and spillover of each firm.

We examine the relationship between sentiment connectedness and stock price crash risk in S&P 500 stocks with several regressions. We find that a firm’s sentiment connectedness is significantly and positively related to crash risk, showing that a firm with higher connectedness in the sentiment network is more vulnerable to crashes. This result demonstrates that stock price is not only related to a firm’s individual sentiment, but also susceptible to sentiment interactions with its neighbors. The relationship between sentiment connectedness and crash risk can be explained in two dimensions. First, a highly interconnected firm may be more influential in spreading sentiment shocks as it can more efficiently reach other members of the network. Second, a highly connected member may be more easily influenced by others, as its network position provides more opportunities to contact peer firms, allowing imitation of behaviors and fostering irrational dynamics. This finding remains robust when considering other determinants of crash risk, i.e., stock price synchronicity, accounting conservatism, extreme market events, and internal corporate governance strength. To address potential endogeneity issues, two-stage least squares estimation and propensity score matching are employed. After considering the impact of peer effect, i.e., the peer companies’ crash, and high-frequency trading, the relationship between sentiment connectedness and crash risk remains robust.

To demonstrate the advantage of considering the sentiment connectedness between firms, we compare the sensitivity of firms’ individual sentiment and the sentiment connectedness to crash risk under different market conditions. Considering that spillover is more likely to occur when sentiment becomes extreme [40,41], we divide the whole sample into periods of heightened and diminished investor sentiment based on the median of the firm’s investor sentiment. Our result shows that both the firm’s sentiment and the sentiment connectedness are significantly and positively related to crash risk across the full sample, indicating that sentiment and the connectedness may increase the firm’s crash risk. However, a firm’s individual sentiment is not a significant driver of crash risk when market sentiment is at a high level or low level. In contrast, sentiment spillover remains a significant influence on crash risk. This can be explained by the fact that during periods of extreme sentiment, investor sentiment can be fully reflected by the market [6], so the impact of sentiment fluctuations on stock price volatility is relatively small. Moreover, when market sentiment is heightening and diminishing, investors generally hold optimistic or pessimistic views, making them more likely to ignore negative information or risk factors, thereby lacking sensitivity to potential crash risks. However, when investor sentiment spreads and propagates through communication or media coverage, other investors may also be affected, thereby exacerbating fluctuations and changes in market sentiment. This indicates that investor sentiment connectedness has stronger explanatory power for crash risk than a firm’s individual sentiment.

We further show potential channels that affect the relationship between sentiment connectedness and firm crash risk. Moderating effect analysis suggests that stock price synchronicity amplifies the effect of network connections on crash risk, but accounting conservatism lessens it. This is because stock price synchronicity increases information asymmetry and raises investors’ sensitivity to bad news. However, accounting conservatism restricts executives’ opportunities to conceal bad news, increases information transparency, and reduces investor sensitivity [42,43,44]. Furthermore, through heterogeneity analysis, we show that extreme risk events and poor company governance can improve the effect of sentiment connectedness on crash risk.

The contribution of this paper can be concluded from three aspects. First, the sentiment connectedness among firms is studied, and a corresponding sentiment spillover network is constructed. While many prior studies focus on the economic impacts of investor sentiments (see, e.g., Kumar and Lee (2006) [45], De et al. (1990) [46], and Han (2008) [47]), they overlook the interplay of individual sentiments. This paper investigates the sentiment interactions between firms using the network structure and measures the investor sentiment connectedness with a novel aggregated centrality index. Our sentiment network shows that the contagion of investor sentiment is not merely driven by the firm financial characteristics such as size, which means that the sentiment network can capture the interaction between investors. Importantly, the structure of the sentiment network also reflects the market dynamics, as the network is more heterogeneous in crisis years. These findings show that the sentiment network is useful and important in describing the financial market.

Second, this paper expands the investigation of firm crash risk by considering the effect of sentiment connectedness among firms. Prior research has predominantly concentrated on analyzing fundamental factors such as managerial characteristics and company fundamentals (see, [48,49,50], etc.). However, the presence of financial market low-efficiency and stock price synchronicity indicates that the risk of stock market crashes is frequently influenced by factors beyond company fundamentals [14,51]. This point has been validated by numerous real-life crisis events, for example, the bank panic following the failures of *Silicon Valley Bank* and *Signature Bank*. Despite the impacts of investor sentiment being shown in some studies, the spillover and network structure of sentiment are neglected. In this paper, we show that the connectedness of firm sentiment, which is beyond fundamental factors, may cause a firm crash. Our study enriches the reason for firm crash risk.

Finally, this paper demonstrates that a firm’s sentiment connectedness holds greater explanatory power regarding crash risk than its individual sentiment. Our study integrates investor interaction networks into the research on investor sentiment, revealing that these networks significantly influence investor behavior. Social networks accelerate information dissemination, enhancing its impact. Anticipating this spread helps us better understand market dynamics. Moreover, differences in how investors interpret information within social networks trigger trading activities (Peng and Zhang, 2024 [52]). Using social network analysis, we can study investor behavior and its effects on trading volume and market liquidity.

The remaining sections of this paper are structured as follows. Section 2 presents definitions and methods pertinent to empirical analysis. Section 3 discusses the data source and fundamental conditions. Section 4 presents the empirical findings. Lastly, Section 5 summarizes the conclusions.

## 2. Methodology

### 2.1. Sentiment Spillover Network

#### 2.1.1. Sentiment Spillover Network Construction

This paper uses the turnover rate as the measure of investor sentiment. The turnover rate can represent the heterogeneity of investor’s beliefs in the market and capture the liquidity of individual stocks. A higher turnover rate also often means more hot market and high investor sentiment [53]. In particular, when irrationally optimistic investor sentiment rises due to short-selling constraints, rational investors will actively trade to profit from their divergence in views [6]. We calculate the turnover rate as the ratio of reported share volume to circulating shares of stock *i* on day *t*.

In this paper, we construct sentiment spillover between firms using the nonlinear Granger network method proposed in [22]. For a sample of the turnover of S&P 500 component stocks over the period from January 2006 to December 2021, the procedure includes the following steps:

First, we filter linear causality using the VAR model, and use VAR residuals to carry out the nonlinear Granger causality test concretely for stationary time series $x_{t}$ and $y_{t}$. Similar to [54], we establish the following linear bivariate VAR:

(1) $$\begin{array}{c} x_{t}=a_{10}-b_{10}y_{t}+b_{11}x_{t-1}+b_{12}y_{t-1}+\varepsilon_{xt},\\ y_{t}=a_{20}-b_{20}x_{t}+b_{21}x_{t-1}+b_{22}y_{t-1}+\varepsilon_{yt}, \end{array}$$

where $x_{t}$ and $y_{t}$ are stationary times series and the optimal lag order of the VAR(n) according to the Akaike information criterion. Then, we obtained the residual sequences $\varepsilon_{xt}$ and $\varepsilon_{yt}$.

Second, a BDS test is performed on the VAR residuals from the VAR linear filter [55], which is based on the correlation dimension of the nonlinear structure in the test time series. If the test negates the null hypothesis, the tested time series is nonlinear.

Finally, we test the nonlinear Granger relation between any two nodes as the edge of the network. The null hypothesis ($H_{0}$) is that $X_{t}$ is not Granger causing $Y_{t}$. In practice, conditional independence is tested using finite lags $l_{x}$ and $l_{y}$

(2) $$Y_{t+1}\left|\left(X_{t}^{l_{x}};Y_{t}^{l_{y}}\right)∼Y_{t+1}\right|Y_{t}^{l_{y}},$$

where $X_{t}^{l_{s}}=\left(X_{t-l_{x}+1},\cdots X_{t}\right)$ and $Y_{t}^{l_{s}}=\left(Y_{t-l_{y}+1},\cdots Y_{t}\right)$, $l_{x},\;l_{y}>1$. We suppose that $Z_{t}=Y_{t+1}$ and $z_{t}\in Z_{t}$. Therefore, U=(X,Y,Z) denotes a three-variate random variable, distributed as $U_{t}=\left(X_{t},Y_{t},Z_{t}\right)$. Then, Equation (2) can be rewritten as $\frac{f_{X,Y,Z}\left(x,y,z\right)}{f_{X,Y}\left(x,y\right)}=\frac{f_{Y,Z}\left(y,z\right)}{f_{Y}\left(y\right)}$.

Following [54], the null hypothesis can be implied as

(3) $$q\equiv E[f_{X,Y,Z}\left(x,y,z\right)f_{Y}\left(y\right)-f_{X,Y}\left(x,y\right)f_{Y,Z}\left(y,z\right)]>0.$$

Then, the test statistic for nonlinear Granger causality is

(4) $$T_{n}\left(\varepsilon_{n}\right)=\frac{n-1}{n(n-2)}\sum_{i=1}^{n}\left[{\tilde{f}}_{X,Y,Z}\left(x_{i},y_{i},z_{i}\right)f_{Y}\left(y_{i}\right)-{\tilde{f}}_{X,Y}\left(x_{i},y_{i}\right){\tilde{f}}_{Y,Z}\left(y_{i},z_{i}\right)\right],$$

where *n* is the size of sample, showing the number of nodes in the network, and ${\tilde{f}}_{U}\left(u_{i}\right)$ is the local density estimator of *U* at $u_{i}$ [56]. The test statistic $T_{n}$ can be interpreted as an average over local BDS test statistics [55], for the conditional distribution of *X* and *Z*, given $Y=y_{i}$. In this case, the test statistic $T_{n}$ is asymptotically normally distributed.

We obtain the asymmetric matrix $E={\left(e_{ij}\right)}_{n\times n}$ through a nonlinear Granger causality test to reveal the direction of sentiment spillover between every pair of firms (i,j), that is,

(5) $$e_{ij}=\left\{\begin{array}{cc} 1, & if\;i\;nolinear\;Granger\;causes\;j\;when\;i\neq j;\\ 0, & otherwise. \end{array}\right.$$

We follow [22] and suppose that for each i∈(1,2⋯n), $e_{ii}=0$, which excludes the connections between the node and itself.

This paper uses the network density d(G) to measure the connections of investor sentiment in the market. The density d(G) characterizes how closely stock nodes are in the network. The larger the density of the network, the more closeness between stock nodes [57]. The density of the network can be calculated as follows:

(6) $$d\left(G\right)=\frac{2L}{n(n-1)},\;$$

where *L* represents the actual number of edges connected to the network, and *n* denotes the number of nodes. The value range of network density is [0,1]; the network density is one when the network is fully connected.

#### 2.1.2. Measuring the Firm’s Sentiment Connectedness: An Entropy Weight Method

This paper considers the sentiment connectedness of firms based on the sentiment spillover network. A sentiment spillover network refers to the concept that sentiment can spread from one individual to another within a social network. It suggests that the sentiment expressed by one individual can influence their neighbor’s sentiment state. According to Matthew et al. (2008) [29], in a financial network where nodes represent firms and linkages are financial connections such as director relationships [24] and obligation connections [23], firms should have high connectedness if they are (1) linked to many other firms, meaning they have many directed connections with other firms; (2) close to other individuals, meaning they have less distance and can spread information to other firms; (3) on the path connecting any other pairs of firms, showing that these firms can transmit information easily; and (4) linked to other firms that have many connections, indicating that these firms are linked to other authority nodes and can spread information effectively. Therefore, this paper considers four primary types of sentiment network centralities, i.e., degree centrality, closeness centrality, betweenness centrality, and eigenvector centrality.

The degree centrality (DC) is a relatively intuitive index, that is, the nodes with more connected edges can spread to more firms. Suppose that $k_{i}$ is the number of neighbors of node *i* and *n* is the total number of nodes in the network, then the degree centrality of node *i* can be measured as

(7) $$DC_{i}=k_{i}/\left(n-1\right).$$

The closeness centrality denotes the average shortest spillover distance between a node and all other nodes. The closeness centrality ($CC_{i}$) of node *i* is calculated as

(8) CCi=(n−1)(n−1)∑i≠jdij∑i≠jdij,

while *n* is the number of in the network and $d_{ij}$ is the shortest distance from *i* to *j*.

The betweenness centrality (BC) measures how many spillover paths a node lies on, indicating the importance of a node in spreading through different parts of the network, that is,

(9) $$BC_{i}=\sum_{j=1}^{n}\frac{\langle n_{ij}\rangle }{n_{ij}},$$

where $\langle n_{ij}\rangle$ denotes the number of shortest edges from *i* to *j* and $n_{ij}$ is the number of edges from *i* to *j*.

Eigenvector centrality ($EC_{i}$) indicates the importance of a node in a spillover network; specifically, the connectedness of a node is related to the number and power of the adjacent nodes [58]. If $x_{i}$ is the important measure of node *i*, the centrality is calculated in the following text:

(10) $$EC_{i}=c\sum_{j=1}^{n}a_{ij}x_{j},$$

while *c* is a proportional constant. $a_{ij}=1$ if vertices i and j are connected by an edge, and $a_{ij}=0$ if they are not.

To mitigate the bias and violation in measuring a firm’s connections, we further quantify a firm’s sentiment connectedness by aggregating these centrality indexes. Suppose that $C_{ij}$ represents the centrality measurement *j* for company *i*, that is,

(11) $$\left[\begin{array}{cccc} C_{11} & C_{12} & C_{13} & C_{14}\\ C_{21} & C_{22} & C_{23} & C_{24}\\ \vdots & \vdots & \vdots & \vdots \\ C_{n1} & C_{n2} & C_{n3} & C_{n4} \end{array}\right]$$

Then, the entropy of each factor can is calculated as

(12) $$E_{j}=-\frac{1}{\ln n}\sum_{i=1}^{n}p_{ij}\ln p_{ij},$$

where $p_{ij}=\frac{C_{ij}}{\sum_{i=1}^{n}C_{ij}}$, then the weight of centrality index *j* can be calculated as

(13) $$\omega_{j}=\frac{1-E_{j}}{\sum_{k=1}^{4}1-E_{k}},$$

The weight ω shows the importance of each centrality in a firm; therefore, we can measure the firm investor sentiment connectedness as

(14) $$FirmSentix_{i}=\sum_{j=1}^{4}\omega_{j}C_{ij},$$

where $C_{j}$ denotes the network centrality and $\omega_{j}$ is the weight obtained from the entropy method [39]. Our sentiment connectedness index can show the full spillover and connection information of a firm in the sentiment network, and implies that a firm with a higher FirmSentix has more sentiment spillover in the network.

### 2.2. Model Identification and Variable Measurements

This paper chooses a fixed-effect model to identify the impact of sentiment connectedness on crash risk. The model can be expressed as

(15) $$\begin{array}{ccc} CRASH_{i,t} & = & \gamma_{0}+\gamma_{1}CRASH_{i,t-1}+\gamma_{2}FirmSentix_{i,t}+\gamma_{3}DTURN_{i,t}+\gamma_{4}ROE_{i,t}\\ & & +\gamma_{5}MB_{i,t}+\gamma_{6}LEV_{i,t}+\gamma_{7}SIZE_{i,t}+\gamma_{8}Opaque_{i,t}+\theta_{t}+\mu_{i}+\varepsilon_{i,t}, \end{array}$$

where $CRASH_{i,t}$ represents the crash risk for firm *i* in year *t*. $FirmSentix_{i,t}$ is the sentiment connectedness of firm *i* at year *t*. $\theta_{t}$ and $\mu_{i}$ are year and firm dummies.

Following Callen and Fang (2013) [59], Equation (15) also includes various multidimensional economic variables as control variables, which are the logarithmic total assets (SIZE), book-to-market ratio (BM), asset–liability ratio (LEV), and return on net assets (ROE), as well as company-specific factors such as information opacity indicators (Opaque) and investor divergence strength (DTRUN). Additionally, to mitigate the potential lag effect caused by crash risk, we incorporate the lag phase of crash risk as an additional control variable. As the trading and sentiment may be influenced by high-frequency trading (HFT), we also consider the HFT market size (HFT) in this paper. The definitions of the variables used in this study are provided in Appendix A.

According to Kim et al. (2014) [60], we consider two crash risk measures. We calculate firm-specific weekly returns ($W_{j,\tau }$) by taking the natural logarithm of one added to the residual earnings from the extended market model regression for each year and firm.

(16) $$r_{j,\tau }=\alpha_{j}+\beta_{1j}r_{m,\tau -2}+\beta_{2j}r_{m,\tau -1}+\beta_{3j}r_{m,\tau }+\beta_{4j}r_{m,\tau +1}+\beta_{5j}r_{m,\tau +2}+\varepsilon_{j,\tau },$$

where $r_{j,\tau }$ is the return on stock *j* in week τ. $r_{m,\tau -2}$, $r_{m,\tau -1}$, $r_{m,\tau }$, $r_{m,\tau +1}$, and $r_{m,\tau +2}$ represent the market return in week τ−2, τ−1, τ, τ+1, and τ+2, respectively. The lead and lag terms for the market index return are included to allow for non-synchronous trading [60].

The firm-specific weekly returns for firm *j* in week τ are computed by $W_{j,\tau }=1+\ln\left(1+\varepsilon_{j,\tau }\right)$, where ε is the residual in Equation (16). Then, we select negative conditional return skewness (NCSKEW) as a measure of each company’s stock price risk, figured by taking the negative of the third moment of firm-specific weekly returns for each year and dividing it by the cubic standard deviation of firm-specific weekly returns. In particular, we compute the (NCSKEW) for each firm *j* in year *t* as

(17) $$NCSKEW_{j,t}=\frac{-T{\left(T-1\right)}^{3/2}\sum_{\tau =1}^{T}W_{j,\tau }^{3}}{\left(T-1\right)\left(T-2\right){\left(\sum_{\tau =1}^{T}W_{j,\tau }^{2}\right)}^{3/2}},$$

where *T* is the number of weekly returns during year *t*. A higher NCSKEW indicates that a stock is more prone to crashes, and vice versa. Another measure of crash risk is the down-to-up volatility (DUVOL). To calculate this measure, we follow [12] and separate the weeks with firm-specific weekly returns above the annual average (up weeks) from those with firm-specific returns below the annual mean (down weeks). We then compute the standard deviation for each subset of weeks. It is calculated as

(18) $$DUVOL_{j,t}=\ln\frac{\left(n_{u}-1\right)\sum_{d\in down}W_{j,d}^{2}}{\left(n_{d}-1\right)\sum_{u\in up}W_{j,u}^{2}},$$

where $W_{j,\tau }$ represents the weekly returns to stock *i* during period τ. $n_{u}$ is the number of up weeks and $n_{d}$ is the number of down weeks.

## 3. Data

Our research sample includes the daily turnover of stocks comprising the S&P 500 index from 4 January 2006 to 31 December 2021. Following Huynh et al. (2021) [61], we apply specific criteria to select stocks for our study: (i) exclusion of stocks suspended for more than 180 consecutive trading days; (ii) removal of unlisted stocks at the beginning of the study period; and (iii) elimination of delisted stocks at the end of the sample period. Consequently, our analysis focuses on a total of 372 constituent stocks of the S&P 500. All the data used in this study are from the WIND database.

Similar to Baker et al. (2012) [20], we choose the turnover rate of a firm as our primary sentiment proxy. Baker and Stein (2004) [51] assert that short-sale constraints cause noise traders to preferentially transact when feeling optimistic rather than pessimistic. Specifically, irrational investors’ optimism is reflected in an ascending turnover ratio, while their pessimism corresponds to a descending turnover ratio. This theoretical link between sentiment and trading activity has also been supported empirically [50]. The turnover ratio is also well suited for capturing volatility and the mean–variance relationship due to its daily availability. As a widely accessible metric computed directly from stock exchange data, the turnover ratio explicitly measures sentiment in equity markets rather than other financial markets [62].

Table 1 reports the descriptive statistics for key variables in this study. Sample firms exhibit a mean turnover of 0.9509, median of 0.6812, and maximum value of 110.7406, suggesting significant volatility in market sentiment within the U.S. equity market. The average crash risk measure ($NCSKEW_{i,t}$) stands at 0.5845, aligning with prior empirical findings. These results highlight the elevated crash risk among S&P 500 firms, underscoring their relevance for in-depth investigation.

## 4. Empirical Results

### 4.1. Sentiment Spillover Network Among S&P 500 Stocks

**Figure 1:**
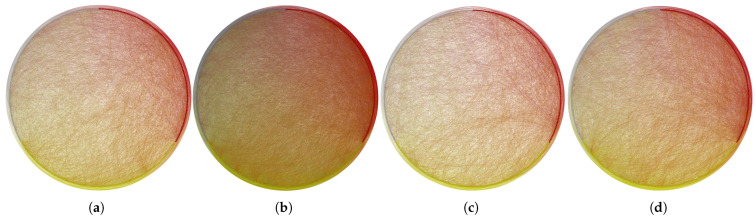
The structural characteristic analysis of sentiment spillover networks. (**a**) shows the sentiment spillover network in 2007; (**b**) shows the sentiment spillover network in 2008; (**c**) shows the sentiment spillover network in 2019; (**d**) shows the sentiment spillover network in 2020.

We construct a sentiment spillover network between S&P 500 stocks from 2006 to 2021. Figure 1 shows the network in the years 2007, 2008, 2019, and 2020. In each network, nodes represent firms and directed arrows show the spillover between firms.

**Figure 2:**
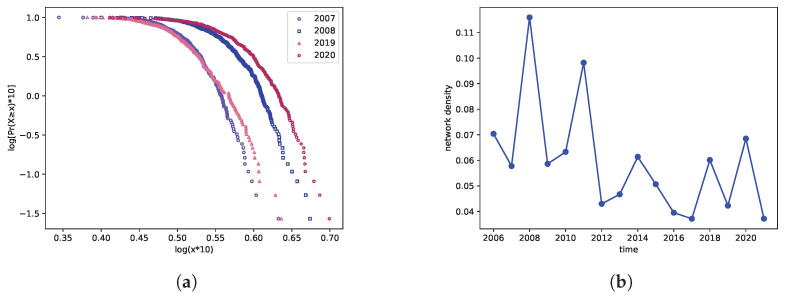
The structural characteristic of sentiment spillover networks. (**a**) is the cumulative distribution of sentiment connectedness for the years 2008, 2009, 2019, and 2020; (**b**) is the yearly network density.

Figure 2a depicts the cumulative distribution of sentiment connectedness for the years 2008, 2009, 2019, and 2020. The results reveal a highly heterogeneous distribution pattern, where most network nodes exhibit limited sentiment linkages while a small subset demonstrates significant connectivity. Notably, crisis periods (2008 and 2020) display more pronounced heterogeneity and connectedness compared to non-crisis years. This finding is visually supported by the flatter distribution curves observed during crisis periods, as opposed to the steeper distributions seen in 2007 and 2019.

It is important to note that our sentiment networks are driven by exogenous investor behavioral factors rather than company fundamentals. This distinguishes our study from previous research on financial networks that focused on business connections [24,31] or price linkage [22,28]. From Figure 1, we find that the connectedness of investor sentiment cannot be solely attributed to company financial characteristics. For example, within the sentiment network, *Amazon* is influenced by numerous other firms, whereas *JP Morgan Chase* and *Goldman Sachs*, despite being globally recognized as important financial institutions, receive fewer sentiment connectedness from other firms. This phenomenon highlights the potential for a firm to establish connections through its influence on another firm’s sentiment, even without directly affecting ownership or asset flows. It suggests that sentiment connectedness is not solely determined by the prominence of a firm.

Figure 2b examines the temporal evolution of network density to characterize systemic interconnectedness. Notably, crisis periods exhibit pronounced spikes in sentiment network density. During the 2008 subprime crisis, density rose sharply from 0.0578 to 0.1159, reaching the highest value observed over the entire sample period. Following this peak, density subsequently declined steadily until 2011, when the U.S. sovereign debt crisis triggered another significant increase. Conversely, network density experienced rapid contraction during post-crisis market recovery phases, reflecting reduced information diffusion across market participants.

Furthermore, during subsequent crises such as the Sino-US trade war in 2018 and the COVID-19 crisis in 2020, the network density reached varying degrees of peaks. Hence, we can infer that network density possesses a certain level of identification for financial crises. This is because an increase in network density signifies heightened stock correlations, thereby increasing the likelihood of risk transmission.

### 4.2. Quantifying the Impact of Sentiment Spillover on Crash Risk

We examine the impact of the sentiment connectedness on stock price crash risk using several panel regression models. To test for possible collinearity problems between explanatory variables, a correlation analysis is carried out. The results show that the correlation coefficients between variables are less than 0.2, indicating that the regression model does not have collinearity problems. Furthermore, since the empirical literature on forecasting crash risk is relatively new, our analysis may omit some crash determinants correlated with other included variables from the regressions. To mitigate potential problems that can arise from correlatedly omitted variables, we attempt to use a fixed effect model. Before it, we perform the Hausman test, which shows that the model is reasonable [63,64]. The regression results are rendered in Table 2.

As illustrated in Table 2, the regression coefficient reveals a positive association between sentiment connectedness and stock price crash risk, suggesting that sentiment connectedness contributes to the amplification of crash risk. This might be because that a firm with higher connectedness has a greater capacity and efficiency to receive and spread extreme sentiment. The general transmission of the signal promotes forming an irrational investment atmosphere, which increases information asymmetry and the possibility of covering “bad news”, raising the volatility of stock prices and crash proneness [65]. Our results also have economic meanings, that is, a change of one standard deviation in FirmSentix results in an approximately 11.4%(1.2895×0.0518/0.5845) increase in NCSKEW.

### 4.3. Robust Test

#### 4.3.1. Endogeneity: Instrumental Variables

To eliminate the potential effects of endogeneity on our results, this study selects the instrumental variables (IVs) of independent variables. Similar to Hao and Xiong (2021) [66], we select the average value of investor sentiment connectedness to other firms in the identical state (Instrument_S) and the average value of investor sentiment connectedness to other firms in the identical industry (Instrument_I), that is,

(19) $$Instrument\_S=\sum_{j\in state}FirmSentix_{j}/\left(n-1\right),$$

(20) $$Instrument\_I=\sum_{j\in industry}FirmSentix_{j}/\left(m-1\right),$$

where $\sum_{state}FirmSentix$ is the sum of sentiment connectedness in the same state, and *n* is the number of firms in the same state. $\sum_{industry}FirmSentix$ is the sum of sentiment connectedness in the same industry, and *m* is the number of the firms in the same industry.

Furthermore, since there may be a mutual causal relationship between the current stock price crash risk and sentiment connectedness, we add the current stock price crash risk as a control variable to the first-stage regression. To avoid the impact of the company’s stock price crash risk from the previous year on the current stage, we add one-year-lagged stock price crash risk to the second stage. The results of the two-stage regression process are shown in Table 3.

The first regression stage is shown in the first column of Table 3. Sentiment connectedness is positively correlated with these two instrumental variables at the 1% significance level. The results of the second regression stage are shown in the second column of Table 3. The investor sentiment connectedness is positively correlated with stock price crash risk (β = 29.885), and the significance level is 1%. Therefore, the endogeneity analysis supports our main results.

#### 4.3.2. Endogeneity: Propensity Score Matching

Given the inherent challenges in quantifying and controlling certain country-level and firm-level factors, we employ the propensity score-matched method to re-evaluate our model. This method allows us to account for the differences in firm characteristics between high and low sentiment connectedness. Specifically, we classify firms with sentiment connectedness above the median as having high sentiment connectedness, and vice versa. In the first stage, we include the firm-level control variables of high sentiment connectedness as outlined in Equation (15), and calculate the propensity score for each sample. The next step involves matching each firm year in the high-sentiment-connectedness group with the nearest propensity score in the low-sentiment-connectedness group. Panel A presents the results of the first-stage regression, and Panel B provides the estimation of the relationship between sentiment connectedness and crash risk in Table 4. Notably, these results remain unchanged when compared to our main regression in Table 2.

#### 4.3.3. The Effect of Network Centrality Measures

To further detect robustness of the results, we also test the relationship between connectedness and crash risk using other network connection measures. Since the importance of the sentiment spillover network measures the spillover of a firm, we consider the impact of LeadeRank centrality (LR), K-coreness centrality (KC), and PageRank centrality (PR) on firm crash risk.

The estimated results are illustrated in Table 5. We show that the relationship between sentiment spillover network centrality indexes (LR, KC and PR) and crash risk remains positive, showing that a firm located in a more important position has a higher crash risk. This result confirms the robustness of our main regression in Table 2. Since the importance of a company in the sentiment spillover network gives it a high ability to acquire and propagate information in a certain group or different groups, this raises the herd effect and irrational behavior of investors, causing crash risk [29].

#### 4.3.4. The Peer Effect

We further examine the peer effect on stock crash risk. Extant literature (e.g., Bustamante and Frésard (2020) [67]; Chen et al. (2019) [68]) has documented that firms often mimic peers’ decisions based on observable characteristics, underscoring the role of peer influence in shaping stock price dynamics. Specifically, Mugerman et al. (2014) [69] and Kaustia et al. (2015) [70] highlight that firms may influence others through peer effect. To isolate the standalone effect of sentiment connectedness from peer-induced crash risk, we incorporate peer companies’ crash risk as a control variable, which is defined as the average crash risk of all firms in the same industry:

(21) $${\mathrm{Peer}}_{i}=\frac{\sum_{j\in industry}{NCSKEW}_{j}}{m-1},$$

where *m* denotes the total number of firms in the industry. Column (1) of Table 6 reports the results after controlling for peer crash risk. Notably, FirmSentix remains significantly negative in predicting crash risk, reinforcing the robustness of our baseline findings.

#### 4.3.5. The Effect of High-Frequency Trading

As competition intensifies, high-frequency trading (HFT) has rapidly evolved. HFT uses computer algorithms to exploit short-term price patterns, involving extremely fast order placement, execution, and cancellation. It is fully automated, characterized by very high-volume trading and short-term holding periods, typically within microseconds. HFT has caught the attention of academics, practitioners, and market regulators, who are increasingly concerned about its impact on market quality [71]. Moreover, many studies point out that HFT would spreading investor sentiment, altering the trading dynamic [72]. Zhang (2010) posits that HFT has pernicious effects on U.S. capital markets, making stock prices overreact to fundamental news [73]. Jarrow and Protter (2012) suggest that HFT trading may drive market prices from fundamental values and cause greater volatility [74].

In this section, we consider the effect of HFT trading on firm crash risk. We consider the HFT market size from 2006 to 2021 as a control variable (the HFT market size data were obtained from https://www.ibisworld.com/united-states/market-size/high-frequency-trading/4740/, accessed on 10 February 2025). Column (2) of Table 6 shows the relationship between FirmSentix and firm crash risk. After controlling the HFT market, we still find a negative impact of sentiment connectedness on crash risk. This result suggests that while HFT may impact market trading and investor sentiment, the contagion of investor sentiment remains a key factor influencing crash risk.

### 4.4. Comparison of Firms’ Individual Sentiment and the Sentiment Connectedness

We compare the impact of sentiment and its connectedness on crash risk in this part. Different from firms’ individual sentiment, connectedness focuses on the interdependence and co-movement among individual sentiments, which are more likely to happen during extreme conditions [40,41]. Therefore, we consider the influence of sentiment and its connectedness when sentiment is surging or declining.

We divide the whole sample into first and last 50% quantile sub-samples. Contrary to the whole sample, the upsurge and downsurge sentiment sub-samples show extreme conditions of firm sentiment, in which the connectedness is more likely to be contagious. We test the sentiment and its connectedness in the whole sample, the upsurge sub-sample and the downsurge sub-sample. We find that both the firm’s individual sentiment and the sentiment spillover network are significantly related to crash risk in whole samples. However, under extreme market conditions, groups (1) and (2) in Table 7 demonstrate that sentiment connectedness has a significant impact on crash risk. More importantly, we find that the influence of sentiment on crash risk is different in three sub-samples, and it is significant only when using a whole sample, which shows that sentiment is related to crash risk under normal market conditions. It reflects that the influence of investor sentiment in the upsurge and downsurge stages on crash risk mainly stems from sentiment connectedness.

These results show that sentiment connectedness is a better predictor of crash risk, especially during market extremes, which highlights the need to include sentiment connections in research models. During severe market stress, investor emotions become highly divided, causing investors to act similarly. This process quickly spreads sentiment shocks across the market [46,75]. As a result, the effect of sentiment changes on stock prices becomes weaker. However, when investor sentiment spreads through communication channels or media reports, it can infect other investors, increasing market sentiment volatility and making stock price swings bigger [76].

### 4.5. Potential Channel Analysis

So far, our findings show a substantial positive correlation between sentiment connectedness and the likelihood of a stock market meltdown. In pursuit of a deeper understanding of the underlying potential channel that links sentiment connectedness and stock price crash risk, a moderating effect model is established as follows:

(22) $$\begin{array}{cc} NCSKEW_{i,t}=\alpha + & \beta_{1}FirmSentix_{i,t}+\beta_{2}FirmSentix_{i,t}*Mod_{i,t}+\beta_{3}DTURN_{i,t}\\ & +\beta_{4}MB_{i,t}+\beta_{5}ROE_{i,t}+\beta_{6}SIZE_{i,t}+\beta_{7}LEV_{i,t}+\beta_{8}Mod_{i,t},\\ & +\beta_{9}NCSKEW_{i,t}+\beta_{10}Opaque_{i,t}+\theta_{t}+\mu_{i}+\varepsilon_{i,t} \end{array}$$

where $Mod_{i,t}$ represent the two mediators of synchronicity ($Synch_{i,t}$) and accounting conservatism ($Cscore_{i,t}$). The variable $Synch_{i,t}$ indicates the co-movement of firms and markets, which is used as a proxy for the quantity of firm-specific information contained in stock price for firm *i* in year *t*, and the variable $Cscore_{i,t}$ indicates the accounting conservatism for firm *i* in year *t*, which uses the index developed by Khan and Watts (2009) [77].

#### 4.5.1. Moderation Effects of Stock Price Synchronicity

Building on the theoretical framework, our analysis reveals that firms with higher sentiment connectedness can rapidly access more impactful sentiment information compared to their peers. Specifically, hub companies mitigate stock price crash risk by reducing stock price synchronicity, enhancing information transparency, and curbing investor irrationality during extreme sentiment periods [44]. These mechanisms motivate the inclusion of stock price synchronicity as a mediating variable in our analysis. The empirical results are presented in Table 8.

To uncover the moderating impact of stock price synchronism, we provide an interacting variable of the sentiment connectedness and stock price synchronicity ($FirmSentix_{i,t}*Sync_{i,t}$). Column (2) reveals that stock return synchronicity amplifies the influence of the sentiment connectedness on stock price crash risk. This may be due to the fact that companies with high stock price synchronization move in lockstep with the market, which reduces the transparency of firm-specificity information and raises the blindness in investing, and raises the influence of investor sentiment.

#### 4.5.2. Moderation Effects of Accounting Conservatism (Cscore)

As executives may overstate performance or conceal bad news for compensation or career incentives [49], firms accessible to extreme sentiment sooner face governance disadvantages during periods of heightened emotion. According to [60,78,79], the supervision of irrational behavior by accounting proxy can reduce the concealment of “bad news” by company managers, thereby reducing the stock price risk of a company caused by the externalities effect of investors for extreme sentiment.

We seek to determine whether sentiment connectedness with a high Cscore increases the effect of information asymmetry on the risk of price collapses [77]. Therefore, we provide a interacting variable of the sentiment connectedness variable and Cscore ($FirmSentix_{i,t}*Cscore_{i,t}$). As illustrated in Table 8, Column (3) presents the results when sentiment connectedness interacts with accounting conservatism (Cscore), and the significant negative coefficient of $FirmSentix_{i,t}*Cscore_{i,t}$ indicates that accounting conservatism can mitigate the effect of the sentiment connectedness on stock price crash risk. This could be because firms with high accounting conservatism have better ability to control excessive managerial self-interest, improve information symmetry, and lower illogical investment risk. Therefore, the accounting conservatism reduces the impact of irrational sentiment.

To examine whether sentiment connectedness exacerbates the effect of information asymmetry on crash risk [77], we introduce an interaction term between sentiment connectedness ($FirmSentix_{i,t}$) and accounting conservatism ($Cscore_{i,t}$). As shown in Table 8, Column (3) reports the results for this interaction model. The significantly negative coefficient on $FirmSentix_{i,t}*Cscore_{i,t}$ indicates that accounting conservatism mitigates the impact of sentiment connectedness on stock price crash risk. This finding can be attributed to firms with strong accounting conservatism having better capacity to constrain managerial opportunism, enhance information transparency, and reduce irrational investment behavior. By improving information quality, accounting conservatism thereby weakens the transmission of irrational sentiment across market participants.

### 4.6. Further Analyses

To further explore the positive relationship between sentiment connectedness and stock price crash risk, we conduct sub-sample regressions to examine how ownership structure and financial condition moderate this association after controlling for corporate governance variables.

#### 4.6.1. The Effect of Shareholding Structure

We first examine firms’ shareholding structures as they form the foundation of corporate governance. Prior research has indicated that ownership concentration may help to alleviate agency conflicts between major and minority shareholders by enhancing informational asymmetry within firms [79]. Therefore, we define a high ownership concentration if a firm’s initial shareholder ratio is greater than 25%, and other firms have a low shareholding concentration. The results are presented in Table 9.

Columns (1) and (2) in Table 9 show that sentiment connectedness and crash risk are positively related at the 1% significance level in companies with low shareholding concentrations, while no relationship is observed for firms with high shareholding dispersion. This indicates that a low-concentrated shareholding structure strengthens the influence of spillover networks on firm crash risk. This can be explained by the fact that a low-concentrated structure is not conducive to disseminating firm-specific information, which could increase investor blindness and the probability of irrational behavior [50]. Such irrational sentiment may then cause dramatic swings in stock prices, thereby increasing crash risk.

#### 4.6.2. The Effect of Financial Condition

The firm’s financial condition is our second measure of corporate governance. When a firm is in financial trouble, i.e., cash flow is insufficient, its earnings might not meet its investors’ expectations, which would result in a decline in its stock price and firm value [80]. Based on Zang (2012) [81], we choose −2.675 as the boundary of financial distress, that is, above −2.675 indicates financial distress and below −2.675 indicates financial steadiness.

We show the results in Table 9. As illustrated in Table 9, Columns (3) and (4) suggest that the association between sentiment connectedness and crash risk is more prominent in financial-distress firms but not in financially sound firms. This result means that a sound financial condition decreases the effect of spillover networks on firm crash risk. This may be because investors are likely to pay more attention to the behavior of the executives of distressed companies. The management of distressed firms is inclined to manipulate earnings management to cover up the real situation of the firm’s fundamentals when investor sentiment becomes extreme [82]. This increases the opaque in such (distressed) firms, which would increases the crash proneness.

## 5. Conclusions

This paper examines how sentiment connectedness between firms impacts stock price crash risk. We argue that companies with higher connectedness have a higher ability to receive and disseminate sentiment, exacerbating investor’s blindness and irrationality. This can severely propagate behaviors like “herding effects” and “enthusiasm”, ultimately lead to price crashes. Controlling for other determinants, sentiment connectedness incrementally forecasts crash risk above other predictors. Furthermore, sentiment influences on crash risk during upturns and downturns appear driven by connectedness between firms. These findings prove robust to models incorporating synchronicity, accounting conservatism, and governance quality. Two-stage least squares estimation and propensity score matching address endogeneity concerns. We find that stock synchronicity amplifies connectedness’s effects on crash risk, while conservatism mitigates the effect. Finally, we analyze how ownership structure and financial distress moderate the relationship between sentiment connectedness and crash risk. This research sheds light on conditions influencing how sentiment disperses between firms and materializes in stock prices.

We contribute to understanding the role of sentiment connectedness in corporate governance and financial risk. By examining the relationship between firm-level sentiment contagion, accounting practices, and stock returns, we extend prior literature by identifying how sentiment connectivity influences stock price crash risk. Our findings highlight that sentiment spillover networks play a critical role in propagating crash risk, suggesting that these systemic linkages should be incorporated into risk management frameworks. Overall, this research deepens our understanding of how shared investor psychology and information diffusion through financial networks impact market outcomes. To mitigate crash risk, firms and regulators should prioritize monitoring sentiment connectedness alongside traditional risk measures.

Our study combines social networks with investor sentiment, presenting an innovative behavioral finance perspective. Theoretically, it pioneers a new approach to financial market analysis by focusing on investor sentiment interaction. First, investor interaction networks help us understand the relationship between information dissemination and market reactions. While behavioral finance focuses on how investors process information and make decisions, prior studies show that market sentiment and investor behavior are often influenced by external events and collective emotions, as demonstrated by Edmans et al. (2007) [83] and Abudy et al. (2022) [84]. Social networks among investors provide a fast channel for information spread. Investor interaction networks enable public information to be quickly disseminated among investors, significantly enhancing the impact of information release (Cookson et al., 2024 [85]). Therefore, integrating social networks into behavioral finance allows for a more in-depth study of market responses to information.

Second, combining investor micro-behavioral characteristics with network structures helps us understand belief divergence and trading dynamics. In social networks, different investors’ interpretations and belief differences regarding the same information trigger trading behavior (Peng and Zhang, 2024 [52]). Using social network data, we can study the extent of belief divergence and its impact on trading volume and market liquidity. Through interactions in social networks, investors engage in social learning and continuously update their beliefs. This learning process is influenced by factors such as social network structure, information quality, and individual cognitive biases.

Lastly, social networks may spread misinformation or trigger overreactions, causing market prices to deviate from their true value, which is crucial for behavioral finance research. In practice, combining behavioral finance with social network analysis offers regulatory bodies enhanced market monitoring tools. By examining information flow and investor sentiment on social networks, regulators can promptly detect illegal activities like market manipulation and misinformation, ensuring market order. Moreover, this research aids in crafting sound policies to promote the healthy use of social networks in finance and protect investor interests.

This study offers crucial advice for investors. First, firms with high sentiment connectedness are more prone to stock price crashes. Retail investors can use sentiment connectedness metrics (e.g., FirmSentix) to spot centrally positioned companies in sentiment networks, which may experience sharp price swings during market mood shifts. Our research also highlights the need for investors to be mindful of inter-firm sentiment spillovers. This implies that selecting unconnected stocks for sector-based asset allocation can reduce sentiment-linked risks. In short, our study shows that market sentiment contagion can trigger crash risks. Therefore, investors might need to use technical tools to monitor market sentiment or add low-correlated assets to their portfolios.

## Figures and Tables

**Table 1:** Descriptive statistics of all variables.

Variable	Mean	Median	Std.Dev	25%	75%
Turnover	0.9509	0.6812	1.2750	0.4537	1.0668
FirmSentix	0.3488	0.3448	0.0518	0.3139	0.3815
NCSKEW	0.5845	0.3264	1.4818	−0.2292	1.0159
DUVOL	0.3730	0.2500	1.0784	−0.2560	0.8145
SYNC	0.9430	0.7374	1.4826	0.1605	1.3944
Opaque	0.0852	0.0690	0.0640	0.0396	0.1125
Cscore	0.1024	0.0598	0.5051	−0.0251	0.1407
DTRUN	−0.0175	−0.2066	9.8562	−2.9545	2.6064
ROE	25.9194	14.6409	887.9436	8.7548	24.1418
LEV	0.6290	0.6195	0.2739	0.4758	0.7622
SIZE	23.4522	23.4596	1.9902	22.4874	24.4137
MB	0.3072	0.0053	1.3380	−0.3439	0.7442
HFT	21.7037	7.5198	20.9112	6.6078	39.5887

Note: This table reports the mean, median, standard deviation, and upper and lower quartiles of the data used for panel regression and network construction. All variables in this table are defined in Appendix A.

**Table 2:** The regression of sentiment connectedness and crash risk.

Variable	Column (1)	Column (2)	Column (3)	Column (4)
	${\mathrm{NCSKEW}}_{i,t}$	${\mathrm{NCSKEW}}_{i,t}$	${\mathrm{DUVOL}}_{i,t}$	${\mathrm{DUVOL}}_{i,t}$
$FirmSentix_{i,t}$		1.2895 *** (2.82)		0.5287 * (1.82)
$DTURN_{i,t}$	0.0031 (1.39)	0.0032 (1.41)	0.0025 (1.62)	0.0025 (1.63)
$ROE_{i,t}$	1.08 × 10^−5^ (1.33)	1.21 × 10^−5^ (1.53)	9.46 × 10^−6^ *** (2.72)	9.98 × 10^−6^ *** (2.88)
$MB_{i,t}$	−0.3556 *** (−26.93)	−0.3547 *** (−26.83)	−0.3461 *** (−32.36)	−0.3458 *** (−32.30)
$LEV_{i,t}$	0.1128 (0.72)	0.1107 (0.72)	0.1021 (0.92)	0.1013 (0.93)
$SIZE_{i,t}$	0.0350 (1.18)	0.0328 (1.11)	0.0233 (1.25)	0.0224 (1.20)
$Opaque_{i,t}$	0.6218 (1.28)	0.6249 (1.27)	0.3279 (1.00)	0.3393 (1.00)
$NCSKEW_{i,t-1}$	−0.1096 *** (−8.66)	−0.1073 *** (−8.58)		
$DUVOL_{i,t-1}$			−0.1173 *** (−11.04)	−0.1167 *** (−10.99)
Constant	0.0173 (0.02)	−0.3993 (−0.56)	−0.1391 (−0.30)	−0.9980 (−0.80)
Firm	Yes	Yes	Yes	Yes
Year	Yes	Yes	Yes	Yes
*F*	145.92	141.38	161.73	153.33
$Adj.R^{2}$	0.242	0.243	0.295	0.298
Obs.	5564	5564	5564	5564

Note: This table presents the key results from the estimation of the relation between sentiment connectedness and stock price crash risk. Columns (1) and (3) are results without the sentiment connectedness variable, and columns (2) and (4) are regression results with the sentiment connectedness. The *t* test values are in parentheses, where *** and * indicate statistical significance at the 1% and 10% levels, respectively. Details regarding each variable used in this study are presented in Appendix A.

**Table 3:** Sentiment connectedness and crash risk: instrument variable method.

Variable	Column (1)	Column (2)
	${\mathrm{FirmSentix}}_{i,t}$	${\mathrm{NCSKEW}}_{i,t}$
$FirmSentix_{i,t}$		29.8850 ***
		(10.28)
$NCSKEW_{i,t-1}$		−0.0970 ***
		(−7.91)
$NCSKEW_{i,t}$	0.0021 **	
	(2.35)	
$Instrumet\_I_{i,t}$	1.1341 ***	
	(7.79)	
$Instrument\_S_{i,t}$	0.1348 **	
	(2.11)	
$DTURN_{i,t}$	−0.0004	0.0105 ***
	(−1.27)	(4.30)
$ROE_{i,t}$	−1.83 × 10^−6^ *	6.54 × 10^−5^ ***
	(−1.68)	(5.22)
$MB_{i,t}$	−0.0013	−0.3050 ***
	(−1.27)	(−19.99)
$LEV_{i,t}$	−0.0018	0.0072
	(−0.20)	(0.04)
$SIZE_{i,t}$	0.0013	−0.0184
	(0.71)	(−0.62)
$Opaque_{i,t}$	0.0057	0.5941
	(0.16)	(1.17)
Constant	−0.0600	−10.9749 ***
	(−0.84)	(−9.48)
Firm	Yes	Yes
Year	Yes	Yes
*F*	30.74	109.82
$Adj.R^{2}$	0.085	0.161
Obs.	5936	5564

Note: This table shows the analysis results of instrumental variables. Column (1) is the result of the first regression stage, and column (2) is the result of the second regression stage. The *t* test values are in parentheses, where ***, **, and * indicate statistical significance at the 1%, 5% and 10% levels, respectively.

**Table 4:** Propensity score matching results.

Panel A: First-stage propensity score matching	
Variable	High sentiment connectedness
$DTURN_{i,t}$	0.0091 ***
	(5.32)
$ROE_{i,t}$	−2.846 × 10^−5^
	(−1.14)
$MB_{i,t}$	−0.1492 ***
	(−10.97)
$LEV_{i,t}$	−0.0923
	(−1.47)
$SIZE_{i,t}$	−0.0411 ***
	(−3.73)
$Opaque_{i,t}$	0.0344
	(0.13)
$NCSKEW_{i,t-1}$	−0.0322 ***
	(−2.78)
Constant	1.1358 ***
	(4.35)
Year	Yes
Obs.	5564
$Pseudo\;R^{2}$	0.003
Panel B: Sentiment connectedness and crash risk	
Highsentimentconnectedness	6.8832 ***
	(9.08)
Controls	Yes
Obs.	2878
$Adj.R^{2}$	0.155

Note: This table reports the matching method of sentiment connectedness and crash risk propensity score. Panel A is the estimation result of the Probit model in the first stage. The dependent variable of the first stage is high sentiment connectedness. If the connectedness is higher than the sample median, this indicator variable is one, and otherwise, it is zero. We regressed high sentiment connectedness for firm features and use the estimated coefficients from the first-stage model to calculate the propensity score for each observation in the sample. We then matched each firm year in the high-connectedness group with the firm year in the low-connectedness group and obtained the closest propensity score. Panel B reports the OLS results of propensity score matching samples to test the intermediate relationship between sentiment connectedness and crash risk. The values of the *t* test are in parentheses, with *** indicating statistical significance at the 1%.

**Table 5:** Results with alternative network centrality measures.

Variable	Column (1)	Column (2)	Column (3)	Column (4)
	${\mathrm{NCSKEW}}_{i,t}$	${\mathrm{NCSKEW}}_{i,t}$	${\mathrm{DUVOL}}_{i,t}$	${\mathrm{DUVOL}}_{i,t}$
$LD_{i,t}$		0.0735 **		
		(2.48)		
$KC_{i,t}$			0.0253 ***	
			(13.54)	
$PR_{i,t}$				23.4394 **
				(2.17)
$DTURN_{i,t}$	0.0031	0.0030	0.0137 ***	0.0030 **
	(1.39)	(1.32)	(3.94)	(2.32)
$ROE_{i,t}$	1.08 × 10^−5^	1.23 × 10^−5^	1.91 × 10^−5^ *	1.20 × 10^−5^
	(1.33)	(1.49)	(1.76)	(1.46)
$MB_{i,t}$	−0.3556 ***	−0.3546 ***	−0.3541 ***	−0.3547 ***
	(−26.93)	(−26.86)	(−30.39)	(−26.86)
$LEV_{i,t}$	0.1128	0.1068	0.2083	0.1070
	(0.72)	(0.69)	(1.33)	(0.69)
$SIZE_{i,t}$	0.0350	0.0351	−0.0032	0.0319
	(1.18)	(1.17)	(−0.11)	(1.17)
$Opaque_{i,t}$	0.6218	0.6153	0.8447 *	0.6911
	(1.28)	(1.26)	(1.71)	(1.42)
$NCSKEW_{t-1}$	−0.1096 ***	−0.1074 ***	−0.1043 ***	−0.1076 ***
	(−8.66)	(−8.58)	(−7.99)	(−8.59)
Constant	0.0173	−0.0628	0.0258	−1.0572
	(0.02)	(−0.09)	(0.03)	(−1.49)
Firm FE	Yes	Yes	Yes	Yes
Year FE	Yes	Yes	Yes	Yes
Observations	5564	5564	5564	5564
Adj. $R^{2}$	0.242	0.243	0.170	0.243
*F*-stat	145.92	142.55	271.67	142.80

Note: This table presents regression results using alternative network centrality measures. Columns (2)–(4) report results using LeaderRank, K-coreness, and PageRank centrality, respectively. All specifications include firm and year fixed effects. Robust t-statistics in parentheses. *** *p* < 0.01, ** *p* < 0.05, * *p* < 0.1.

**Table 6:** The effect of sentiment network under HFT and peer effect.

Variable	Column (1)	Column (2)
	${\mathrm{NCSKEW}}_{i,t}$	${\mathrm{NCSKEW}}_{i,t}$
$FirmSentix_{i,t}$	1.0501 **	1.3399 ***
	(2.04)	(2.93)
Peer		0.7710 *
		(−1.95)
HFT	2.4320 **	
	(2.30)	
$DTURN_{i,t}$	0.0032 **	0.0040 **
	(2.41)	(2.37)
$ROE_{i,t}$	1.21 × 10^−5^	1.18 × 10^−5^
	(1.53)	(1.53)
$MB_{i,t}$	−0.3547 ***	−0.2916 ***
	(−26.83)	(−26.70)
$LEV_{i,t}$	0.1107	0.1168
	(0.72)	(0.70)
$SIZE_{i,t}$	−0.0329 *	0.0220
	(1.80)	(1.03)
$NCSKEW_{i,t-1}$	−0.6241 *	−0.2012 **
	(1.82)	(1.76)
$Opaque_{i,t}$	−1.1056 ***	−1.1056 ***
	(−7.59)	(−7.59)
Constant	−0.395	−1.408
	(−0.56)	(−0.26)
Firm FE	Yes	Yes
Year FE	Yes	Yes
*F*-stat	128.03	142.80
Adj. $R^{2}$	0.215	0.245
Obs.	5564	5564

Note: This table presents results controlling for HFT market size (Column 1) and peer effects (Column 2). All specifications include firm fixed effects. Robust t-statistics in parentheses. *** *p* < 0.01, ** *p* < 0.05, * *p* < 0.1.

**Table 7:** Comparison of sentiment connectedness and sentiment.

Variable	Group (1) Upsurge		Group (2) Downsurge		Group (3) Whole	
	${\mathrm{NCSKEW}}_{i,t}$	${\mathrm{NCSKEW}}_{i,t}$	${\mathrm{NCSKEW}}_{i,t}$	${\mathrm{NCSKEW}}_{i,t}$	${\mathrm{NCSKEW}}_{i,t}$	${\mathrm{NCSKEW}}_{i,t}$
$FirmSentix_{i,t}$	1.6873 ** (2.55)		1.3522 * (1.75)		1.2895 *** (2.82)	
$Sentiment_{i,t}$		−0.0044 (−0.08)		0.0253 (−0.17)		−0.1064 ** (−2.47)
$DTURN_{i,t}$	0.0042 (1.50)	0.0042 (−1.65)	0.0090 (1.13)	−0.0039 (−1.05)	0.0032 (1.39)	0.0012 (−0.46)
$ROE_{i,t}$	2.77 × 10^−5^ *** (4.44)	2.53 × 10^−5^ *** (−3.8)	−0.0008 (−0.32)	8.11 × 10^−6^ −0.74)	1.21 × 10^−5^ (1.53)	3.14 × 10^−6^ (0.27)
$MB_{i,t}$	−0.4242 *** (−17.70)	−0.4253 *** (−17.69)	−0.3307 *** (−16.15)	−0.3251 *** (−20.49)	−0.3547 *** (−26.83)	−0.3559 *** (−26.94)
$LEV_{i,t}$	0.1311 (0.61)	0.1215 (−0.56)	0.5017 (1.35)	0.0596 (−0.27)	0.1107 (0.72)	0.1241 (−0.8)
$SIZE_{i,t}$	0.0513 (1.52)	0.0526 (−1.57)	0.1533 (1.61)	−0.0401 (−0.41)	0.0328 (1.11)	0.0282 (−0.94)
$Opaque_{i,t}$	0.3163 (0.46)	0.3162 (−0.46)	1.5577 (1.47)	1.0433 (−1.55)	0.6249 (1.27)	0.6331 (−1.29)
$NCSKEW_{i,t-1}$	−0.0913 *** (−4.77)	−0.0924 *** (−4.78)	−0.1539 *** (−5.31)	−0.1203 *** (−7.47)	−0.1073 *** (−8.58)	−0.1073 *** (−8.50)
Constant	−0.8977 (−1.05)	−0.3054 (−0.37)	−4.1146 * (−1.86)	1.6847 (−0.76)	−0.3993 *** (−0.56)	0.2521 (−0.35)
Firm	Yes	Yes	Yes	Yes	Yes	Yes
Year	Yes	Yes	Yes	Yes	Yes	Yes
*F*	65.67	69.02	35.74	96.33	141.38	146.01
$Adj.R^{2}$	0.232	0.4021	0.243	0.257	0.243	0.244
Obs.	2760	2760	2790	2790	5564	5564

Note: This table is the result of both sentiment connectedness and sentiment on crash risk. *High* (*Low*) denotes that sentiment is high (low); 50% sample denotes that the first 50% of the sample is classified as the high-sentiment stage, and the second 50% is classified as the low-sentiment stage. As a whole, *Whole* denotes the effect of the sentiment or sentiment connectedness on stock price. The *t* test values are in parentheses, where ***, **, and * indicate statistical significance at the 1%, 5%, and 10% levels, respectively.

**Table 8:** The effect of sentiment network on crash risk under different conditions.

Variable	Column (1)	Column (2)	Column (3)
	${\mathrm{NCSKEW}}_{i,t}$	${\mathrm{NCSKEW}}_{i,t}$	${\mathrm{NCSKEW}}_{i,t}$
$FirmSentix_{i,t}$		1.1584 **	1.3399 ***
		(2.49)	(2.93)
$Sync_{i,t}$		−0.1346 **	
		(−2.57)	
$FirmSentix_{i,t}\times Sync_{i,t}$		0.4429 **	
		(2.05)	
$Cscore_{i,t}$			0.3603 *
			(1.80)
$FirmSentix_{i,t}\times Cscore_{i,t}$			−0.1777 *
			(−1.94)
$DTURN_{i,t}$	0.0031	0.0046	0.0064 **
	(1.38)	(1.60)	(2.33)
$ROE_{i,t}$	1.08 × 10^−5^	1.18 × 10^−5^ *	7.45 × 10^−6^
	(1.33)	(2.00)	(1.11)
$MB_{i,t}$	−0.3556 ***	−0.3426 ***	−0.3510 ***
	(−26.93)	(−27.61)	(−26.61)
$LEV_{i,t}$	0.1128	0.2268	0.1268
	(0.72)	(1.49)	(0.81)
$SIZE_{i,t}$	0.0350	0.1130 ***	0.0922 **
	(1.18)	(2.70)	(2.33)
$NCSKEW_{i,t-1}$	−0.1096 ***	−0.1036 ***	−0.1076 ***
	(−8.66)	(−8.79)	(−8.46)
$Opaque_{i,t}$	0.6218	0.7911 *	0.7309
	(1.28)	(1.64)	(1.49)
Constant	0.0173	−2.4345 **	−1.8162 *
	(0.02)	(−2.47)	(−1.93)
Firm FE	Yes	Yes	Yes
Year FE	Yes	Yes	Yes
*F*-stat	145.92	140.23	132.01
Adj. $R^{2}$	0.242	0.272	0.260
Obs.	5564	5564	5564

Note: This table reports the moderating effect of stock synchronicity and accounting conservatism on the relationship between sentiment connectedness and crash risk. Column (1) excludes sentiment connectedness; Column (2) includes stock synchronicity interaction; Column (3) includes accounting conservatism interaction. t-statistics in parentheses. ***, **, * denote significance at the 1%, 5%, and 10% levels.

**Table 9:** Heterogeneity analysis.

Variable	Shareholding Concentration		Financial Condition	
	(1) High	(2) Low	(3) Non-Distressed	(4) Distress
	${\mathrm{NCSKEW}}_{i,t}$	${\mathrm{NCSKEW}}_{i,t}$	${\mathrm{NCSKEW}}_{i,t}$	${\mathrm{NCSKEW}}_{i,t}$
$FirmSentix_{i,t}$	3.7140 (1.57)	1.1481 ** (2.46)	1.4280 (1.64)	1.2249 ** (2.25)
$DTURN_{i,t}$	0.0014 (0.38)	0.0098 *** (4.82)	0.0036 (1.01)	0.0064 * (1.93)
$ROE_{i,t}$	−0.0002 (−0.11)	1.06 × 10^−5^ (1.34)	3.96 × 10^−5^ (1.30)	9.18 × 10^−6^ (1.16)
$MB_{i,t}$	−0.4375 *** (−6.75)	−0.3497 *** (−26.03)	−0.4078 *** (−14.56)	−0.3416 *** (−23.59)
$LEV_{i,t}$	−0.9383 ** (−2.20)	0.1748 (1.08)	0.2227 (0.97)	0.0371 (0.16)
$SIZE_{i,t}$	0.7077 (1.54)	0.0763 *** (2.64)	0.1063 (0.80)	0.0912 ** (2.20)
$Opaque_{i,t}$	−0.8956 (−0.41)	0.6353 (1.26)	0.9240 (0.91)	0.5222 (0.93)
$NCSKEW_{i,t-1}$	−0.1807 *** (−3.15)	−0.1013 *** (−7.93)	−0.1582 *** (−7.03)	−0.0903 *** (−6.18)
Constant	4.7837 (0.93)	−1.490 ** (−2.08)	−2.3645 (−0.74)	−1.6573 (−1.69)
Firm	Yes	Yes	Yes	Yes
Year	Yes	Yes	Yes	Yes
*F*	168.90	144.95	138.91	115.72
$Adj.R^{2}$	0.217	0.211	0.211	0.246
Obs.	285	5265	1215	4335

Note: This table reports the relationship between sentiment connectedness and crash risk under different subsamples. Columns (1) and (2) report regression results for concentrated and dispersed equity samples. Columns (3) and (4) report regression results for financially sound and unstable samples. Year_Dummy represents a dummy variable about time. The *t* test values are in parentheses, where ***, **, and * indicate statistical significance at the 1%, 5%, and 10% levels, respectively.

## Data Availability

The raw data supporting the conclusions of this article will be made available by the authors on request.

## Appendix A. Variable Measurement

**Variable**	**Definition**
NCSKEW	The negative coefficient of skewness, calculated by taking the negative of the third moment of firm-specific weekly returns for each year and dividing it by the cubic standard deviation of firm-specific weekly returns. See Equation (7).
DUVOL	The other measurement of crash risk; we separate all the weeks with firm-specific weekly returns above the annual average (up weeks) from those with firm-specific returns below the annual mean (down weeks) and compute the standard deviation for each sub-sample. See Equation (8).
Zscore	The financial condition estimated following Li et al. (2017) [82]. See Appendix B for a more detailed explanation and calculate it with Equation (A2).
Cscore	The conservatism score estimated following Khan and Watts (2009) [77]. See Appendix B for a more detailed explanation and calculate it with Equation (A1).
Synch	Measure of stock return synchronicity developed by Morck et al. (2000) [86]. See Appendix B for a more detailed explanation and calculate it with Equations (A3) and (A4).
SIZE	The natural logarithm of the company’ s total assets for each sample year.
LEV	It refers to the book value of all liabilities divided by total assets at the end of a fiscal year.
ROE	A ratio representing the efficiency of the use of shareholders’ funds.
MB	The market-to-book ratio of firm *i* in year *t*, that is, (market price at the end of fiscal year × number of shares outstanding + net asset value per share × number of non-tradable outstanding shares)/book value of equity.
Opaque	A measure of financial reporting opaqueness whose calculation is the 3-year moving sum of the absolute value of annual performance-adjusted discretionary accruals.
DTURN	It is a proxy for the level of investor disagreement whose calculation is the average monthly share turnover over the current fiscal year minus the average monthly share turnover over the previous year, where monthly share turnover is calculated as the monthly share-trading volume divided by the number of shares outstanding over the month.
HFT	The HFT market size in the U.S. from the ISIB World website; the HFT is measured by one thousand billion dollars.

## Appendix B. Corporate Governance and Synchronicity

### Appendix B.1. Measurement of Firm-Specific Accounting Conservatism (Cscore)

We measure the degree of accounting conservatism for each sample and each firm using the firm-year conditional conservatism measure Cscore developed by Khan and Watts (2009) [77]. Concretely, to acquire the Cscore measure, we obtain the coefficients that are collated with conservatism by estimating the following regression, that is, $\gamma_{1}$, $\gamma_{1}$, $\gamma_{1}$, and $\gamma_{1}$ at time t:

(A1) $$\begin{array}{cc} Y_{i}=\beta_{1}+ & \beta_{2}\times D_{i}+R_{i}\times \left(\mu_{1}+\mu_{2}\times Size_{i}+\mu_{3}\times MB_{i}+\mu_{4}\times Leverage_{i}\right)\\ & +D_{i}\times R_{i}\times \left(\gamma_{1}+\gamma_{2}\times Size_{i}+\gamma_{3}\times MB_{i}+\gamma_{4}\times Leverage_{i}\right)\\ & +(\delta_{1}\times Size_{i}+\delta_{2}\times MB_{i}+\delta_{3}\times Leverage_{i}+\delta_{4}\times D_{i}\times Size_{i}\\ & +\delta_{5}\times D_{i}\times BM_{i}+\delta_{6}\times D_{i}\times Leverage_{i})+\varepsilon_{i}, \end{array}$$

where $Y_{i}$ is measured as $EPS/P_{i,t-1}$, with $P_{i,t-1}$ is the stock price at the beginning of time *t*, and EPS is the earnings per share measured by operating profit measured by the number of stocks outstanding. $R_{i}$ is the buy-and-hold yield, adjusted for the corresponding market return, from the fifth month after the end of fiscal year t to the fourth month of year t + 1, $R_{i}=\sum_{t,5}^{t+1,4}(1+r_{i,t})-\sum_{t,5}^{t+1,4}\left(1+r_{m,t}\right)$, where $r_{i,t}$ is the firm monthly return, including dividends, and $r_{m,t}$ is the monthly market value-weighted return. $D_{i}$ is a dummy variable, set to one if $R_{i}<0$ and to zero otherwise. Size is the natural log of the book value of total asserts. MB is the market-to-book ratio, and leverage is the liability-to-asset ratio. Then, we obtain a measure of firm-year conditional conservatism (Cscore), applying the following linear functions:

(A2) $$Cscore_{i}=\gamma_{1}+\gamma_{2}\times Size_{i}+\gamma_{3}\times MTB_{i}+\gamma_{4}\times Leverage_{i},$$

If a firm has a high Cscore, it is considered more conservative.

### Appendix B.2. Measurement of Firm-Specific Financial Distress (Distress)

Many studies use the Z-score defined by Altman (1968) [87], which is usually treated as the most widely used financial distress measure [88]. Zang (2012) [81] modified the Z-score to proxy for a firm’s financial health. Following Zang (2012) [81], we use the modified Z-score to measure financial distress:

(A3) $$ZScore_{i,t}=0.3X_{1}+1.0X_{2}+1.4X_{3}+1.2X_{4}+0.6X_{5},$$

where Distress denotes financial distress, and $X_{1}$ is net profit over total assets. $X_{2}$ is sales over total assets; $X_{3}$ is retained earnings over total assets; $X_{4}$ is working capital over total assets and $X_{5}$ is the market value of equity over total liabilities. If $ZScore_{i,t}$ is larger, the financial condition of firms is better. The $-ZScore_{i,t}$ is little, and the firm is in financial distress.

### Appendix B.3. Measurement of Firm-Specific Stock Price Synchronicity

Stock synchronicity reflects information asymmetry between companies and investors. To measure stock price synchronicity, we first calculate the $R^{2}$ of company i in fiscal year t by using the regression of the market single-factor model [89].

(A4) $$RET_{i,t}=\alpha +\beta MKTRET_{t}+\varepsilon_{i,t},$$

where $RET_{i,t}$ is the return ratio of firm *i* in week *t*, and $MKTRET_{t}$ is the market capitalization weighted return ration in a week. The data selected here are mainly S&P 500 index data weighted by the floating market value. $R_{i,t}^{2}$ for each firm every year is obtained by regression. Since the range of $R_{i,t}^{2}$ is [0,1], following Morck et al. (2000) [86], this paper defines the stock price synchronization index of the firm i at time t.

(A5) $$Synch_{i,t}=\ln\left(\frac{R_{i,t}^{2}}{1-R_{i,t}^{2}}\right),$$

A high $Synch_{i,t}$ indicates that the firm is highly correlated with the market.
